# Origin and evolution of gene families in Bacteria and Archaea

**DOI:** 10.1186/1471-2105-12-S9-S14

**Published:** 2011-10-05

**Authors:** R Eric Collins, Hugh Merz, Paul G Higgs

**Affiliations:** 1 Origins Institute, McMaster University, Hamilton, ON L8S 4L8, Canada; 2 SHARCNET, Laurentian University, Sudbury, ON, P3E 2C6, Canada

## Abstract

**Background:**

Comparison of complete genomes of Bacteria and Archaea shows that gene content varies considerably and that genomes evolve quite rapidly via gene duplication and deletion and horizontal gene transfer. We analyze a diverse set of 92 Bacteria and 79 Archaea in order to investigate the processes governing the origin and evolution of families of related genes within genomes.

**Results:**

Genes were clustered into related groups using similarity criteria derived from BLAST. Most clusters contained genes from only one or a small number of genomes, and relatively few core clusters were found that spanned all genomes. Gene clusters found in larger numbers of genomes tended to have larger numbers of genes per genome; however, clusters with unusually large numbers of genes per genome were found among both narrowly and widely distributed clusters. Larger genomes were found to have larger mean gene family sizes and a greater proportion of families of very large size. We used a model of birth, death, and innovation to predict the distribution of gene family sizes. The key parameter is *r*, the ratio of duplications to deletions. It was found that the model can give a good fit to the observed distribution only if there are several classes of genes with different values of *r.* The preferred model in most cases had three classes of genes.

**Conclusions:**

There appears to be a rapid rate of origination of new gene families within individual genomes. Most of these gene families are deleted before they spread to large numbers of genomes, which suggests that they may not be generally beneficial to the organisms. The family size distribution is best described by a large fraction of families that tend to have only one or two genes and a small fraction of families of multi-copy genes that are highly prone to duplication. Larger families occur more frequently in larger genomes, indicating higher *r* in these genomes, possibly due to a greater tolerance for non-beneficial gene duplicates. The smallest genomes contain very few multi-copy families, suggesting a high rate of deletion of all but the most beneficial genes in these genomes.

## Background

There are now a large number of completely sequenced genomes of Bacteria and Archaea that can be used to study evolution at the whole-genome level. Comparison of sets of genes across genomes reveals that gene content varies quite substantially between even fairly closely related species. For example, the number of genes in a typical genome of *Escherichia coli* and *Shigella* is around 5000, but when 20 of these genomes are compared, there are only around 2000 ‘core’ genes found in all genomes [1]. Similar results are found with *Streptococcus*[2] and *Prochlorococcus*[3]. When diverse groups of genomes are compared, the set of core genes falls to very low numbers. It was estimated that the ‘extended core’ of bacterial genes (i.e. those present in 99% of sequenced genomes) contained only 250 genes [4]. A study aimed at constructing a universal phylogenetic tree [5] found only 31 genes present as clear orthologues in all genomes.

These studies also show that newly sequenced genomes almost always contain genes that have no detectable homologues in existing genomes. The size of the pan-genome (i.e. the set of genes found in at least one of the genomes considered) increases rapidly with the number of genomes studied, and it is often believed that the pan-genome would increase indefinitely if new genomes continued to be added to the data. Rapid gain and loss of genes during genome evolution has important consequences. At the small scale, gain and loss of a few specific genes can explain why some microbial strains are pathogenic and close relatives are not. At the large scale, rapid changes in gene content make the use of phylogenetic methods for Bacteria and Archaea difficult, and given that genes can also be transferred horizontally between unrelated organisms, the very existence of an evolutionary tree for Bacteria and Archaea is questionable [6]. Our aim in this paper is therefore to use quantitative comparative methods across a wide range of genomes in order to better understand the processes giving rise to change in gene content.

Gene duplication has long been recognized as a mechanism by which organisms adapt [7]. A duplicate copy of an existing gene can be maintained in the genome if it evolves a new or more specialized function, or it can be lost through pseudogenization and eventual gene deletion. Families of two or more paralogous genes are common in most genomes, which testifies to the important role played by gene duplication in expansion of genome size and acquisition of new functional genes. Gene families can also arise by evolutionary innovation within a genome, e.g. a new open reading frame could form within a non-coding region, or an existing gene could undergo sequence divergence, insertions/deletions or domain reshuffling to such an extent that it would no longer be classed as part of its original family. The insertion of genes via horizontal gene transfer is also an important mechanism of change in gene content. This could start a new gene family if the recipient genome did not already contain a homologous sequence, or it could add to an existing family.

The relationships among sets of genes within and across genomes are potentially very complex. Similar sequences can arise by speciation (orthologues), duplication (paralogues) or horizontal transfer (xenologues). Disentangling these alternatives would be difficult without manual sequence analysis of each case, and would then be somewhat subjective. In this paper, we deliberately want to keep sequence analysis methods as simple as possible. Therefore, we use simple objective criteria based on BLAST to cluster genes into related groups. The procedure is automated and easily reproducible, and can be repeated many times with different clustering parameters in order to check the sensitivity of the outcome. The procedure does not assign detailed evolutionary relationships among the sequences in each cluster, although it assumes that all sequences in a cluster are descended from a common ancestor by one means or another.

Gene clusters contain genes from one or more genomes. For each cluster, let *k* be the number of genomes that contain at least one gene from this cluster. An important quantity is *G*(*k*), the number of clusters that contain genes from *k* genomes. This is sometimes called the gene frequency spectrum. *G*(*k*) has been measured in several experimental data sets [1-4], and shows a U-shaped distribution, with many genes present in only a small number of genomes, a moderate number of core genes present in (almost) all genomes, and fewer genes at intermediate values of *k.* Modelling the distribution of clusters across genomes is complex because it depends on the branching process that generates the sample of genomes, as well as on the processes of gain and loss of genes from a given genome. We are only aware of one theoretical model that attempts to explain this shape [8].

We will use ‘family’ to denote a group of genes from one genome that are within one cluster. The mean size of families in a cluster, , is usually between 1 and 2, but there are a significant number of clusters with  as large as 10 or more. We show that gene clusters that are more widely spread across genomes tend to have larger gene families. However, clusters with very high  are found at both low and high values of *k.* We also analyze the data by genomes. We define  as the mean family size within a genome. This is again between 1 and 2, but there are a significant number of families as large as 10 or more in most genomes. We show that  and the proportion of large families increase with the size of the genome.

Previous studies have shown that *F*(*n*), defined as the number of families of *n* genes in one genome, is distributed approximately as a power law [9,10], with most families found as singletons and only a few families with large numbers of genes. A number of mathematical models have been constructed to explain this [9,11-18]. It is substantially easier to model *F*(*n*) than *G*(*k*) because it only depends on gain and loss of genes from one genome. These models are formulated in different ways. While there is as yet no consensus on the minimal processes needed to reproduce the gene family size distributions observed in Bacteria and Archaea, there are signs that heterogeneity of evolutionary rates among gene families (e.g. selection acting on multiple classes of genes) may be required to explain the shapes of these distributions [16-18]. Here we infer the presence of multiple classes of genes in the genomes of Bacteria and Archaea using a birth, death, and innovation model [13,19]. Each class represents a set of genes with a different ratio of duplication to deletion rates, perhaps due to the effects of natural selection.

## Methods

### Clustering

Complete nucleic acid and translated proteome sequences were obtained from the NCBI Genomes database for 92 Bacteria from over a dozen phyla (Additional File 1, Table 5), sequenced as part of the Genomic Encyclopedia of Bacteria and Archaea [20], and 79 Archaea (Additional File 1, Table 6). Genes encoded on plasmids associated with each genome sequence were also included, and were treated as part of the genome. BLAST databases were created for each genome with all amino acid sequences encoded by each genome, and all-against-all searches were performed using blastp (v. 2.221). For each pair of genomes, each inter- and intra-genome peptide sequence was used as a query against every other peptide sequence. A direct link was counted between two genes if both BLAST E-values were less than a specified cutoff value, *E_cut_*, and if the length of the locally aligned region found by BLAST was longer than a fraction *f_min_* of the length of both sequences. Sequences were grouped into clusters using the single-link cluster procedure, i.e. sequences are part of the same cluster if there is a direct link between them or if there is a chain of direct links that connects them. These clustering methods, similar to those used by NCBI's blastclust program, were implemented with custom Perl scripts that are available from the authors.

### Definitions

For each cluster, let *g* be the number of genes in the cluster and let *k* be the number of genomes that contain at least one gene from this cluster. The mean family size in the cluster is defined as . Let *G*(*k*) be the number of clusters that contain genes from *k* genomes. The total number of clusters is  and the mean number of genomes contributing to each cluster is . The number of clusters that contain genes from one genome only is *G*(1). These are ‘ORFans’ with no detectable homologues in other genomes. The number of clusters that have at least one gene in every genome is *G_core_.*

For each genome, let *N_genes_* be the total number of genes in the genome, and let *F_tot_* be the total number of families with at least one gene in that genome. The mean family size in the genome is . Let *F*(*n*) be the number of families of *n* genes in a genome. It follows that , and . It is of interest to consider the number of single gene families *F*(1) and the number of large (*n* ≥ 5) families, .

### Quantitative models to predict the distribution of gene family sizes

In this section we describe mathematical models to predict the number of gene families in one genome. We will use lower case *f*(*n*) to indicate the predictions of the model and upper case *F*(*n*) to indicate the observed data. We will use Maximum Likelihood methods to fit *f*(*n*) to *F*(*n*) as closely as possible.

We consider a birth and death process in which the number of genes can increase or decrease by one gene at a time. Let *λ_n_* be the rate of transition from *n* to *n* + 1, and let *δ_n_* be the rate of transition from *n* to *n* – 1. These rates are defined for *n* ≥ 1. The total number of families is not fixed. Let there be a rate *u* of innovation of new gene families with *n* = 1, which could occur via *de novo* origin of a gene within a genome or by acquisition of a gene by horizontal transfer. This is assumed to be independent of the number of families already present in the genome. Families are lost if a deletion occurs in a single gene family, which occurs at rate *δ*_1_*f*(1)*.* In the stationary state, the rates of gain and loss of families are equal; hence(1)

In the stationary state, the rate of change from *n* to *n* – 1 balances the rate of the reverse change. Therefore, *f*(*n*)*δ_n_* = *f*(*n* – 1)*λ_n_*_–1_, from which we obtain(2)

The basic model that we will use in this paper will be termed BDI1, where BDI denotes birth, death and innovation. This is similar to models proposed by Karev et al. [13] and Wòjtowicz and Tiuryn [17]. It is supposed that there is a constant duplication rate *λ* and a constant deletion rate *δ* per gene, so that the birth and death rates are *λ*_n_ = *nλ* and *δ_n_* = *nδ.* From Eq. (2), the stationary distribution for model BDI1 is(3)

where  is the ratio of duplication to deletion rates. It is useful to define the function(4)

The total number of families can then be written as(5)

and the mean family size can be written as(6)

**Figure 1:**
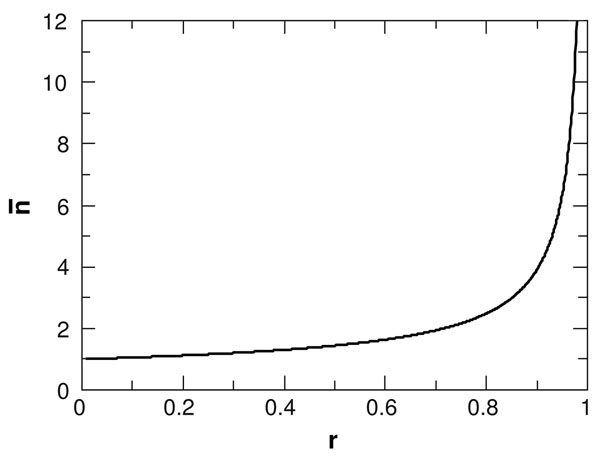
Mean family size  in the BDI1 model as a function of *r*, the ratio of duplication to deletion rates (*λ*/*δ*)*.*

Fig. 1 shows  as a function of *r.* This is close to 1 for small *r* and diverges as *r* approaches 1. Note that the total number of families is proportional to *u*, but the shape of the distribution and the value of  depend only on *r* and not on *u.* The optimal value of *r* can be estimated by Maximum Likelihood methods, as follows.

The proportion of families that are expected to have *n* genes according to the model is(7)

This result has also been given by Wòjtowicz and Tiuryn [17]. The logarithm of the likelihood of observing the data according to the model is(8)

To find *r* for each data set, we used the Nelder-Mead optimization method in R [21] to find the maximum value of ln*L*. It can be shown by substituting the *q_n_* from Eq. (7) and taking the derivative  that the maximum likelihood solution for *r* is such that the mean family size, , given by the model (see Eq. (6)) is equal to the mean family size in the data, *N_genes_*/*F_tot_.* Once *r* is known, it is possible to choose *u*/*δ* so as to make the total number of families predicted by the model (*f_tot_* in Eq. (5)) equal to the observed number, *F_tot_.*

In summary, the BDI1 model has two independent parameters, *r* and *u*/*δ.* These parameters can be chosen so that the mean family size and the total number of families are exactly equal to their values in the data. Having done this, there are no further parameters to adjust the shape of the distribution. It will be shown that the shape is not fitted well by the BDI1 model, so we consider several more general models in the following section.

### More general models

We define model BDIK such that there are *K* classes of genes, each of which behaves as model BDI1. Each class, *k*, has an independent value of *r_k_*. The frequency of families in the genome in class *k* is *ξ_k_*, and these frequencies must sum to 1. Model BDIK thus has 2*K* – 1 free parameters. The stationary *q_n_* distribution for model BDIK is(9)

A multi-class model of this form has also been considered by Wòjtowicz and Tiuryn [17]. A more general birth, death and innovation model (GBDI) proposed by Karev et al. [13] is defined such that *λ_n_* = *λ*(*n* + *a*); *δ_n_* = *δ*(*n* + *b*)*.* It is straightforward to obtain(10)

for *n* > 1, and *q*_1_ can be obtained by normalizing the distribution. This model has three relevant parameters for the shape: *r*, *a*, and *b.* A particular case of this model is where *r* = 1. This has been called the second-order balanced model by Karev et al. [13]. Note that the distribution still converges when *r* = 1, provided *b* >*a.* In this case it can be shown that the distribution is approximately a power law, *q_n_* ~ *n*^–(1+^*^b^*^–^*^a^*^)^ for large *n.*

As it has been argued [9,18] that data of this type can be well represented by a power law, we further consider the simple power law distribution(11)

where the exponent *γ* is the only free parameter.

## Results

### Properties of gene clusters and families

Properties of clusters generated using different values of *E_cut_* and *f_min_* are shown for the Bacteria and Archaea data sets in Tables 1 and 2. Although we changed the *E_cut_* values by many orders of magnitude and varied the minimum length criterion considerably, the most important properties of the clusters did not change very much. In the Bacteria, the total number of clusters *G_tot_* decreased by about a factor of two from the most stringent to the least stringent clustering criteria. The number of core clusters, *G_core_*, increased as the stringency of clustering was reduced, and was always much less than *G_tot_.* In each case, *G*(1) was large and thus ORFan clusters comprised a relatively large fraction of *G_tot_* (typically around 80%). The majority of clusters were only found in a small number of genomes, so that  was small (around 2).

**Table 1:** Properties of gene clusters from Bacteria. Notation is defined in the Methods section.

Clustering parameters		Distribution of clusters across genomes				Distribution of family sizes across genomes				
*E_cut_*	*f_min_*	*G_tot_*	*G*(1)	*G_core_*		*F_tot_*	*F*(1)	*F_large_*		*n_max_*
1e-30	0.8	151049	128755	56	1.78	2928	2682	8.9	1.18	150
1e-30	0.7	143623	121073	68	1.84	2877	2614	9.5	1.20	182
1e-30	0.6	139204	116677	81	1.88	2838	2566	10.5	1.21	189
1e-20	0.8	129357	108673	66	1.97	2765	2470	12.0	1.24	187
1e-20	0.7	119492	98717	79	2.07	2689	2372	13.4	1.28	189
1e-20	0.6	113318	92701	96	2.13	2625	2295	13.2	1.31	311
1e-10	0.8	109361	91573	78	2.13	2537	2199	17.6	1.35	189
1e-10	0.7	96666	79084	98	2.29	2408	2043	19.5	1.42	200
1e-10	0.6	88270	71113	119	2.40	2301	1921	20.1	1.48	452
1e-5	0.8	101042	84903	88	2.15	2365	2016	23.3	1.45	214
1e-5	0.7	86857	71156	107	2.33	2203	1832	25.2	1.55	500
1e-5	0.6	77111	62136	123	2.43	2038	1667	25.9	1.68	791

**Table 2:** Properties of gene clusters from Archaea. Notation is defined in the Methods section.

Clustering parameters		Distribution of clusters across genomes				Distribution of family sizes across genomes				
*E_cut_*	*f_min_*	*G_tot_*	*G*(1)	*G_core_*		*F_tot_*	*F*(1)	*F_large_*		*n_max_*
1e-30	0.8	66742	51882	66	2.38	2036	1867	3.4	1.14	80
1e-30	0.7	63590	48690	77	2.46	2009	1830	3.7	1.16	81
1e-30	0.6	61928	47109	83	2.51	1989	1807	4.0	1.17	90
1e-20	0.8	55885	42059	84	2.72	1946	1738	4.5	1.19	82
1e-20	0.7	51670	37875	105	2.88	1905	1684	5.0	1.22	91
1e-20	0.6	49379	35751	120	2.96	1875	1646	5.2	1.24	106
1e-10	0.8	46648	34801	105	3.01	1798	1548	6.9	1.29	94
1e-10	0.7	41153	29612	132	3.26	1722	1455	9.2	1.35	106
1e-10	0.6	37747	26530	157	3.43	1661	1382	10.6	1.40	156
1e-5	0.8	42850	32258	120	3.05	1677	1411	12.2	1.39	95
1e-5	0.7	36653	26571	140	3.33	1565	1291	16.7	1.48	129
1e-5	0.6	32574	23042	156	3.50	1463	1187	19.1	1.59	197

Properties of the family size distribution also varied to some extent with clustering parameters, although the main points do not depend on the choice of parameters. The quantities *F_tot_*, *F*(1), *F_large_*, and  were calculated for each genome and the figures shown are averages over genomes. The majority of families were single gene families, i.e. *F*(1) was close to *F_tot_*, and  was less than 2 for all choices of the clustering parameters. However, there were significant numbers of large families in all cases, even for the most stringent clustering parameters. This confirms that some genes are indeed frequently duplicated, and large families are not simply an artifact of the clustering technique. The final column of Tables 1 and 2, *n_max_*, is the number of genes in the largest family in any one genome. Although the precise value of *n_max_* depends on the clustering parameters, it is always very much larger than 1. Thus the *F*(*n*) distribution contains a tail of large families that is very different from typical families of only 1 or 2 genes.

The results for the Archaea were similar to those for Bacteria. The parasitic species *Nanoarchaeum equitans* has a genome size less than half the size of any of the other Archaea, and the presence of this species significantly affected the clustering statistics. Therefore the results quoted in Table 2 were calculated for 78 Archaea excluding *N. equitans.*

### Variation of gene family size with genome size

**Figure 2:**
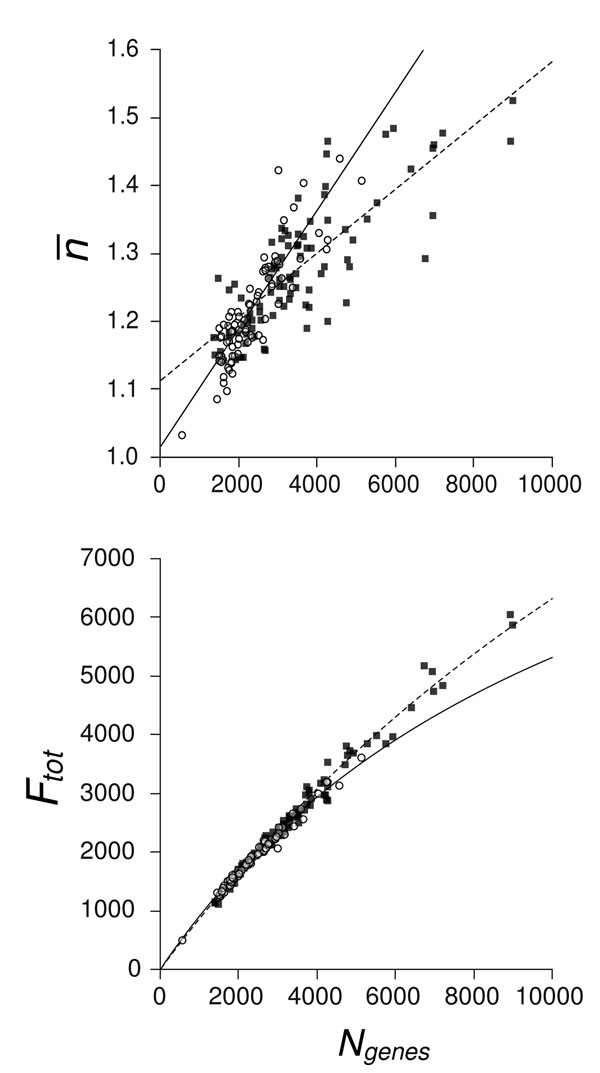
a) Mean family size  against genome size (*N_genes_*) for genomes of 92 Bacteria (■) and 79 Archaea (◦), both clustered at *E_cut_* = 1e-20 and *f_min_* = 0.7. Analysis of variance showed a significant effect of *N_genes_* on  (*F*(1, 167) = 421.54, *p* ≪ 0.001) and a significant interaction between *N_genes_* and taxa group (*F*(1, 167) = 28.27, *p* ≪ 0.001), indicating that the slopes for Bacteria and Archaea were significantly different. Least-squared linear regressions are thus shown separately for Bacteria (dashed line, ) and Archaea (solid line, ). (b) Total number of families (*F_tot_*) against *N_genes_* for Bacteria and Archaea, clustered as above. Lines are the regressions from (a), defined by the relation .

**Figure 3:**
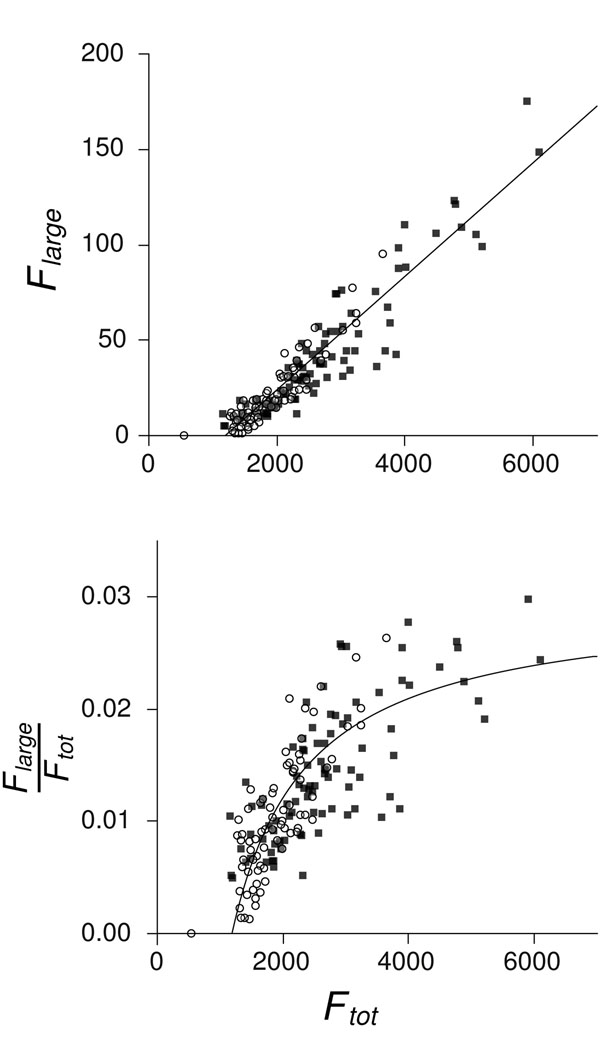
(a) Number of large gene families (*F_large_*) against total number of gene families (*F_tot_*) in the genomes of 92 Bacteria (■) and 79 Archaea (◦), both clustered at *E_cut_* = 1e-20 and *f_min_* = 0.7. Analysis of variance showed a significant effect of *F_tot_* on *F_large_* (*F*(1, 167) = 1181.8, *p* ≪ 0.001) but not of taxa group. The least-squared linear regression is thus shown for the combined Bacteria and Archaea dataset (solid line, *F_large_* = –35.4 + 0.0298 × *F_tot_*); the *x*-intercept for this line is at *F_tot_* = 1186. (b) Fraction of large gene families (*F_large_*/*F_tot_*) against *F_tot_*, clustered as above. Line is the regression from (a), divided by *F_tot_*.

For the more detailed analysis in the remainder of the paper, we used one representative set of clustering parameters: *E_cut_* = 1e-20, *f_min_* = 0.7. The genomes in the two data sets vary in terms of the number of genes they encode. The range in Archaea is 536–5113 genes and in Bacteria, it is 1353–8975 genes. We wished to determine whether there were any variations in the family size distribution correlated with the number of genes. The mean family size per genome  displayed a significant increasing trend with *N_genes_* (Fig. 2a), with Archaea having a significantly greater slope (0.087 per thousand genes) than Bacteria (0.047 per thousand genes). By definition , so *F_tot_* also increases with *N_genes_*, but slightly less than linearly, as shown in Fig. 2b. There was no difference between the number of large gene families in Archaea compared to similarly-sized Bacteria (Fig. 3a). The fraction of large gene families *F_large_*/*F_tot_* increases with *F_tot_* and tends towards a plateau at 2-3% for large genomes (Fig. 3b). The *x*–intercept is at about 1200 families, suggesting a minimum threshold genome size below which large gene families are absent or very rare.

Archaea typically have smaller genomes than Bacteria, but in both taxonomic groups larger genomes tend to have larger gene families. This is not bound to be the case, it could simply have been that larger genomes had a larger total number of families and that the mean family size stayed the same. The fact that the mean family size increases with genome size means that gene duplication plays a significant role in the evolution of genome size. The steeper slope in mean family size within the Archaea may indicate that gene duplication plays a somewhat more prominent role in genome expansion than in Bacteria, or that Archaea preferentially discard duplicate genes during genome contraction. In both Bacteria and Archaea, genome size is influenced both by innovation of new families and by duplication of genes within a family.

### Relationship between gene family size and the number of genomes in which the family is found

**Figure 4:**
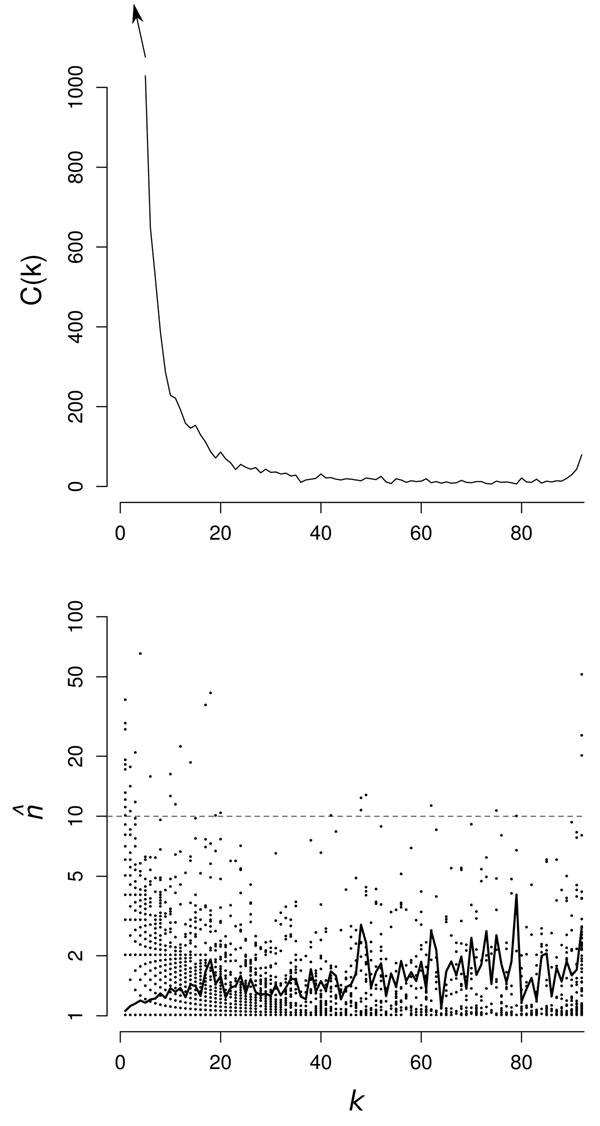
(a) *G*(*k*) for Bacteria clustered at *E_cut_* = 1e-20 and *f_min_* = 0.7. (b)  for Bacteria clustered as above. Symbols indicate clusters (•); black line indicates mean . The most common annotation for each cluster above the gray dashed line is given in Additional File 1, Table 7.

**Figure 5:**
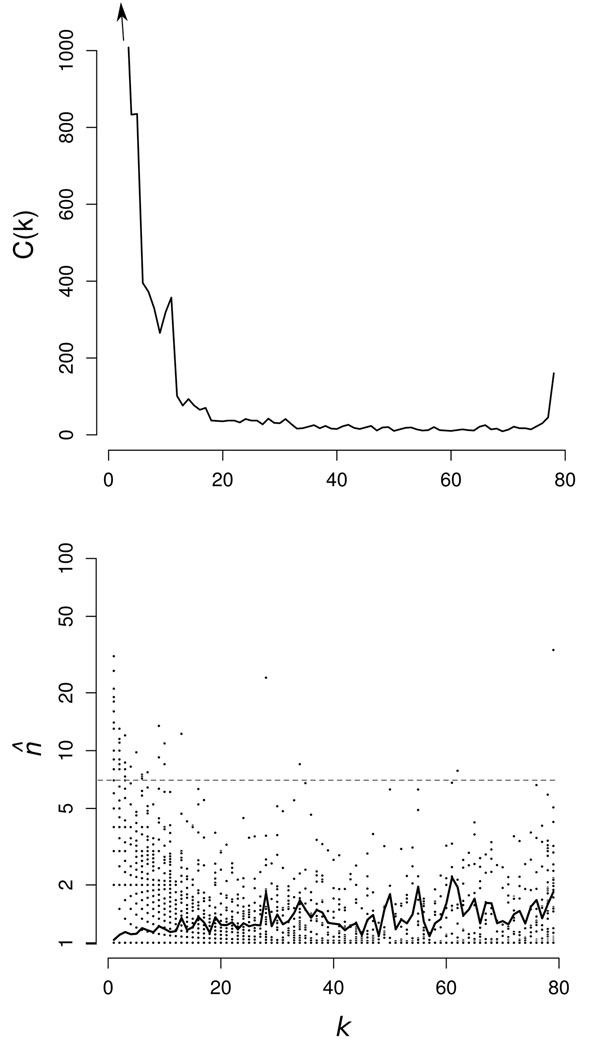
(a) *G*(*k*) for Archaea clustered at *E_cut_* = 1e-20 and *f_min_* = 0.7. (b)  for Archaea clustered as above. Symbols indicate clusters (•); black line indicates mean . The most common annotation for each cluster above the gray dashed line is given in Additional File 1, Table 8.

As defined in the Methods section, *k* is the number of genomes that have at least one gene in a given cluster, and *G*(*k*) is the number of clusters that contain genes from *k* genomes. The *G*(*k*) distributions are shown for Bacteria and Archaea in Figures 4(a) and 5(a). As expected, these are U-shaped distributions with large numbers of clusters in only one or a small number of genomes, fewer clusters with intermediate *k*, and a substantial number in all or almost all genomes. The peak of core genes on the right of the U is relatively small in both datasets because the genomes are very diverse and there are few core genes. We intend to investigate the factors determining the shape of *G*(*k*) in more detail in future work. However, at this point, we wish to focus on the relationship between the mean family size, , within each cluster and the number of genomes, *k*, in which the cluster is found.

In Figures 4(b) and 5(b) we plot  as a function of *k* for the representative data sets. Each point represents one cluster. For the smaller *k* there are many points superimposed, as the  values are always rational fractions. The solid line shows the average  for all clusters with the same *k.* In both cases, this shows a steady increase from very slightly more than 1 for *k* = 1, to about 2 for the core clusters. We conclude that almost all ORFan genes are in single gene families, but genes that are present in larger numbers of genomes have a greater probability to be part of multi-gene families in a given genome, i.e. more widespread genes are more likely to be duplicated. Nevertheless, the mean  is only around 2 or less, even for large *k*, which means that there are many core genes that also exist as single gene families.

It is clear from looking at the points for the individual clusters in Figures 4(b) and 5(b) that there are some clusters with  values much larger than the mean. These unusual clusters are found both at high and low *k.* Clusters with high *k* and high  are presumably core clusters containing large gene families in all genomes, whereas clusters with low *k* and high  are probably the result of recent rapid duplication within a closely related group of genomes. To investigate this in more detail, we looked at the annotation of the genes in the clusters with large . Additional File 1, Table 7 and Additional File 1, Table 8 list the clusters with the largest  in Bacteria and Archaea. The most frequent Genbank annotation of the genes in each cluster is given in the tables. It was found that the core cluster with the maximum  consisted of genes annotated as ABC transporters in both cases. ABC transporters have a wide range of related functions and are known to be widespread in many genomes [22]. We found that there were many separate clusters in our analysis that contained genes annotated as ABC transporters, covering a wide range of substrate specificities. These genes are rapidly evolving and duplicating and appear to be diversifying fast enough to cause the initiation of many new clusters of sequences that are not grouped together according to the criteria we used for clustering.

Additional File 1, Table 7 and Additional File 1, Table 8 also show the high  genes that were present in only a single genome. It was found that many of these genes were transposable elements. As these genes are not widely distributed, we presume they have arisen recently and are duplicating rapidly due to their own transposase activity, even if they are not beneficial to the organism. This contrasts with genes like the ABC transporters, that may not be duplicating particularly rapidly, but which are presumably maintained in the genome in multiple copies because of their beneficial function to the organism. It also appears that there are a few specific genomes in which highly-duplicated, highly-localized genes are over-represented, e.g. among the Bacteria, *Haliangium ochraceum* DSM 14365 and *Bacillus tusciae* DSM 2912, and within the Archaea, *Methanospirillum hungatei* JF-1 and *Sulfolobus solfataricus* P2. In the case of *Haliangium ochraceum*, one of the largest genomes we investigated (9.4 MB), many copies of a cluster of 6 ORFan genes are concentrated into a small region of the genome, flanked variously by mobile elements like transposases and prophage-associated genes, possibly indicating recent duplication events that are out of equilibrium with the rest of the genome.

### Family size distributions

**Figure 6:**
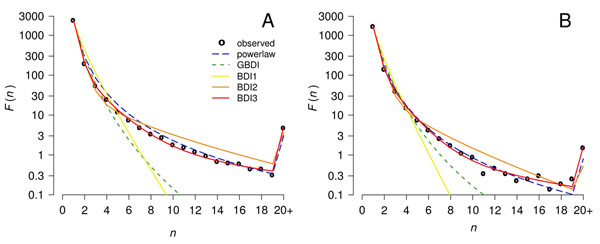
Maximum Likelihood fits of five models to the mean family size distribution *F*(*n*) in genomes from (A) 92 Bacteria and (B) 79 Archaea clustered at *E_cut_* = 1e-20 and *f_min_* = 0.7. BDI3 is the best fit for the constituent genomes. As there are few families larger than *n* = 20, the data point at *n* = 20 shows the sum of all families with *n* ≥ 20, and the theory points at *n* = 20 show the sum of the predicted frequencies of all families with *n* ≥ 20. Hence the apparent spike in these distributions.

The quantitative models defined in the Methods section were fitted to the data using numerical methods to optimize lnL. Figure 6 shows the mean *F*(*n*) distribution for the Bacteria and the Archaea and the fits of the various models. Model BDI1 gave a poor fit to the data, overestimating the frequency of families in the range 2-5 and underestimating the frequency of large families by many orders of magnitude. Models BDI2 and BDI3 gave much better fits; hence it is clearly useful to introduce more than one class of gene. The fit for BDI4 appeared very similar to BDI3 and is not shown. The generalized model, GBDI, fit noticeably better than BDI1, but still considerably underestimated the frequency of large families. The power-law fit performed similarly to models BDI2 and BDI3 at the least restrictive clustering parameters but provided poorer fits as clustering became more strict.

To select the best fitting model in a more principled way, Akaike’s Information Criterion (AIC) was used [23]. This is defined as AIC = 2(–ln*L* + number of free parameters). The model with the smallest AIC is to be preferred because it has a high likelihood without over-fitting the data. The AIC can be applied when fitting each genome individually because the families in one genome are assumed to be independent. However, it cannot be applied to fitting the mean data because families from the same cluster in different genomes are phylogenetically related and are not independent. We applied the AIC analysis to each of the genomes in each of the two sets separately. Model BDI3 was the best fitting model for 85% of the 92 Bacteria genomes under each of the 12 clustering regimes. Model BDI2 was substantially worse, fitting best in only 8% of cases. Model BDI1 was never the best fitting model for any of the Bacteria, nor was the GBDI model. A simple power law was occasionally the best fit (7%). Model BDI4 was always a poor choice by AIC, indicating that the four-class model was over-fitting the data.

The 79 archaeal genomes were most commonly fit best by model BDI3 (60% of clusterings), which was selected much more frequently than BDI2 (18%). Only the reduced genome of *N. equitans* was ever best fit by BDI1. Model BDI4 was very rarely the best fit for Archaea; GBDI was never the best fit. A simple power law was the best fit for a number of Archaea (22% of clusterings).

Having established that the preferred model is BDI3, we show the fitted parameters for this model in Tables 3 and 4 for Bacteria and Archaea, respectively. The column *f_BDI_*_3_ shows the fraction of the individual genomes for which the BDI3 model was the preferred model according to AIC. The *ξ_k_* and *r_k_* columns show the best fit parameters to the average *F*(*n*) for all the genomes in each set. During the fitting procedure, the r values were constrained so that *r*_1_ <*r*_2_ <*r*_3_. The fitted parameters were qualitatively similar independent of clustering parameters chosen or dataset examined. There is always a large fraction of genes in class 1: *ξ*_1_ is between 73 and 85% in Bacteria. The corresponding values of *r*_1_ are around 0.1. From Fig. 1 it can be seen that *n* is very close to 1 in this range of r. Thus, these genes are almost always in single-gene families. Class 2 genes have frequency between 14% and 25%, with *r*_2_ in the range 0.6–0.8. The corresponding *n* is between 1.5 and 2. These genes are also usually in fairly small families. Only 1–3% of genes fall in class 3, and these have *r*_3_ = 0.95 or greater, which corresponds to *n* around 6 or larger. The conclusions for Archaea are similar to those for Bacteria, although the mean value of *r*_3_ is lower than for Bacteria, indicating that the largest families are not quite so large.

**Table 3:** Fitting parameters for gene clusters from Bacteria using the BDI3 model.

*E_cut_*	*f_min_*	*f_BDI_*_3_	*ξ*_1_	*ξ*_2_	*ξ*_3_	*r*_1_	*r*_2_	*r*_3_
1e-30	0.8	0.848	0.767	0.203	0.030	0.102	0.709	0.969
1e-30	0.7	0.837	0.726	0.246	0.028	0.110	0.722	0.978
1e-30	0.6	0.891	0.753	0.233	0.014	0.138	0.804	0.991
1e-20	0.8	0.870	0.771	0.205	0.024	0.086	0.680	0.963
1e-20	0.7	0.837	0.753	0.224	0.023	0.106	0.706	0.972
1e-20	0.6	0.880	0.764	0.222	0.014	0.130	0.761	0.985
1e-10	0.8	0.880	0.823	0.161	0.016	0.082	0.649	0.961
1e-10	0.7	0.891	0.799	0.183	0.018	0.086	0.650	0.962
1e-10	0.6	0.935	0.817	0.172	0.011	0.106	0.710	0.979
1e-5	0.8	0.761	0.850	0.136	0.013	0.064	0.607	0.957
1e-5	0.7	0.826	0.841	0.146	0.013	0.070	0.623	0.962
1e-5	0.6	0.837	0.827	0.159	0.015	0.070	0.614	0.962

**Table 4:** Fitting parameters for gene clusters from Archaea using the BDI3 model.

*E_cut_*	*f_min_*	*f_BDI_*_3_	*ξ*_1_	*ξ*_2_	*ξ*_3_	*r*_1_	*r*_2_	*r*_3_
1e-30	0.8	0.545	0.829	0.160	0.011	0.063	0.544	0.936
1e-30	0.7	0.597	0.810	0.179	0.011	0.064	0.546	0.939
1e-30	0.6	0.646	0.824	0.166	0.010	0.072	0.578	0.951
1e-20	0.8	0.506	0.767	0.218	0.015	0.073	0.553	0.932
1e-20	0.7	0.584	0.749	0.237	0.014	0.075	0.575	0.945
1e-20	0.6	0.582	0.761	0.227	0.012	0.087	0.610	0.954
1e-10	0.8	0.595	0.741	0.241	0.018	0.094	0.649	0.943
1e-10	0.7	0.481	0.716	0.260	0.024	0.103	0.659	0.945
1e-10	0.6	0.532	0.715	0.263	0.022	0.120	0.696	0.954
1e-5	0.8	0.519	0.721	0.231	0.047	0.103	0.661	0.924
1e-5	0.7	0.684	0.687	0.251	0.062	0.108	0.655	0.935
1e-5	0.6	0.873	0.718	0.236	0.046	0.141	0.727	0.961

From these results, it can be seen that in order to explain the large number of single-gene families seen in the data, it is necessary to have a large fraction of genes with relatively small values of r. If r is small, the probability of generating very large gene families is very low. This is the reason why model BDI1 cannot fit the data with only a single class of genes. The data contains a significant number of large gene families. The fitting procedure shows that this is best explained by having a fairly small fraction of genes with an r that is substantially larger than that for the majority of genes.

**Figure 7:**
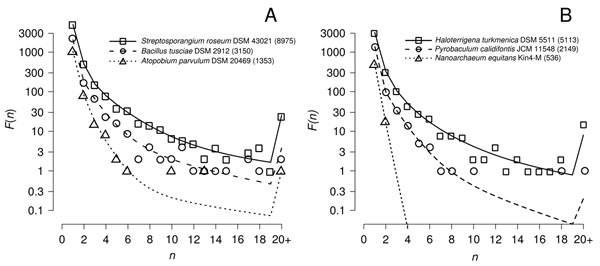
Maximum Likelihood fits of model BDI3 to the family size distribution *F*(*n*) in genomes of (A) 3 Bacteria and (B) 3 Archaea, clustered at *E_cut_* = 1e-20 and *f_min_* = 0.7. The selected taxa are the smallest, median, and largest genomes in the clustered datasets; genome size, as number of protein coding sequences, is shown in parentheses following each taxa name.

As the sizes of genomes differ within each data set, we wish to look at the *F*(*n*) distributions in individual genomes. For both the Bacteria and Archaea, we chose the smallest and largest genomes and a third genome with size close to the median. Figure 7 show the data and the fits to model BDI3 for each of these genomes. The two large size genomes both contain many large families with *n* ≥ 10. The two medium-sized genomes each contain a handful of large families. The smallest bacterium in our data, *Atopobíum parυulum*, with 1353 genes, contains one family of 12 and one of 20, with the rest being 6 or fewer. The smallest archaeon, *N. equitans*, with only 536 genes contains no families with *n* > 2. These show that there are significant differences among organisms in the distribution of family sizes. Large families are rare or absent in small genomes.

## Discussion and conclusions

The aspect of our data that has previously been studied most frequently is the distribution of family sizes within one genome, *F*(*n*)*.* It was shown by Huynen and van Nimwegen [9] that this distribution has a tail of large families that dies away slowly with n; hence it resembles a power law rather than an exponential. This has since been confirmed by many authors and several mathematical models have been proposed to explain it. There are three qualitatively different explanations: (i) family sizes are in constant expansion; (ii) the distribution is stationary and second-order balanced; (iii) the distribution is stationary and there are multiple classes of genes. We will compare these explanations and explain why we favour case (iii).

By ‘stationary’ we mean that the number of families of any given size is not varying with time on average in any one genome. This can occur in the models used here if *r* < 1, i.e. if duplication is less frequent than deletion. Under these circumstances, families do not grow indefinitely and are bound to become extinct eventually. The rate of innovation of new families balances the extinction rate. In contrast, if duplication is more frequent than deletion, then both the total number of genes per genome and the number of genes in individual families increase with time. The models of Yanai et al. [11], Qian et al. [12] and Kamal et al. [16] are examples of explanation (i). These show that if family sizes are in constant expansion, then the shape of *F*(*n*) can converge to a power law after the expansion process has been in operation for some time. However, there is considerable variation in genome sizes in the data, and genomes can increase or decrease in size relatively easily over the time scale of diversification of Bacteria and Archaea. There are many examples of parasitic or endosymbiotic microbes whose genomes have reduced in size in comparison to their closest free-living relatives. It can also be seen from Figure 7 that genomes of different sizes have different family size distributions. Our interpretation is that duplication and deletion rates vary among species. If these rates change along an evolutionary lineage, it will take some time for the family size distribution to come into equilibrium with the new parameters. If we fit a stationary model to data from different genomes, we are assuming that there has been sufficient time for each genome to reach a different equilibrium. This seems reasonable, given that rates of gene gain and loss have been estimated as very high [24], and it seems closer to the truth than the assumption that there is a constant rate of non-stationary expansion.

Explanation (ii) is illustrated by the GBDI model (see Eq. (10)). This model was not a particularly good fit to our data, but we wish to make a few comments about the parameters that emerged when fitting this model. It was found that the value of *r* converged to 1 and the value of *a* converged to 0 for every genome analyzed (boundary conditions were *r* ≤ 1 and *a* > 0). Thus *b* was the only remaining parameter in the fit. The case *r* = 1 is the second-order balanced model of Karev et al. [13], and it is known that the stationary distribution is close to a power law in this case. Nevertheless, we do not find this model very appealing, because it is not intuitively clear why the ratio of insertions to deletions should be tuned exactly to 1 in every case. Also, it is not easy to interpret what the fitting parameters *a* and *b* mean biologically.

In contrast, the BDI models depend on parameters *u*, *λ* and *δ* that have a simple biological interpretation. The distribution for the basic BDI1 model does not resemble a power law, but when different classes of genes are included with different ratios of duplication to deletion, the model fits the data well. This is explanation (iii) for the family size distribution. The simple power law model (Eq. (11)) was included in our analysis as a null model because it has only one parameter, but according to AIC, model BDI3 was a better fit in the majority of cases. The existence of several classes of genes with different rate parameters has also been observed by Wòjtowicz and Tiuryn [17] and Hughes and Liberies [18].

Although we found a minimum of three gene classes were necessary to fit the genomic data, in reality the number of classes could approach the total number of families in a genome. The fact that different types of genes in a genome should have different duplication and deletion rates seems intuitively reasonable because these rates depend on selection. A duplication or deletion arising in an individual cell could be beneficial, neutral or deleterious, and this will influence the likelihood of this variant becoming widespread. Selection parameters are unlikely to be the same for every gene family in the genome.

When fitting the BDI models, we can only estimate the ratio *λ*/*δ*, and not the two separate rates. Self-replicating elements provide one mechanism by which a gene family can be preferentially duplicated over another and thus affect *λ*. A high-*r* class can then represent both self-replicating (high-*λ*) and useful (low-*δ*) gene families. Likewise, low-*r* classes can represent deleterious (high-*δ*) gene families, neutral or ‘junk’ gene families having rapid turnover rates, and core or ‘essential’ gene families for which the loss of the last member is fatal to the organism.

While gene function is not expected to correspond explicitly with the gene classes described above, we might expect some association between functional category and class. For example, genes involved in transcriptional regulation may be found more frequently in multi-gene (high-*r*) families because they can be utilized repeatedly throughout the genome, whereas genes involved in specialized metabolic functions may be found more frequently in single-gene (low-*r*) families. Observations from comparative genomics support this prediction. Molina and van Nimwegen [25] found that the abundance of protein domains associated with ‘regulation of transcription’ scaled quadratically with total number of domains in a genome, whereas ‘metabolic process’ domains scaled less than linearly. In general, different functional categories of genes, each composed of numerous gene families, have been found to vary in representation within genomes and follow scaling laws which may [26,27] or may not [25,28] differ by lineage.

We note that Wòjtowicz and Tiuryn [17] have considered an extra process, occurring at rate *κ*, in which an existing gene mutates to a new kind of gene; thus one family is reduced in size by one and a new family of size one is initiated at the same time. The result of this is that the distribution has the same shape, but depends on the ratio *λ*/(*δ* + *κ*), instead of *λ*/*δ.* This is interesting theoretically, but as we can only measure the ratio from the stationary distribution, it is not helpful to add *κ* to the model.

The next step in analysis of data of this type would be to look at the dynamics of the processes generating the patterns of gene copy numbers across species on a phylogenetic tree. This removes some of the redundancy in parameters, and may allow more detailed conclusions about the mechanisms of gene family evolution than can be obtained from stationary family size distributions. Several models of this type have been investigated [29-31] and we intend to continue on these lines. However, the *κ* process would be difficult to consider in a phylogenetic model because it would mean that changes in copy numbers in different families would not be independent of one another.

There are several aspects of our data that go beyond the family size distribution, and these have been studied less frequently by other authors. One of these is the so-called gene frequency spectrum, *G*(*k*), which is the number of clusters with one or more genes in *k* genomes. This is usually plotted for orthologues only, in which case there is either 0 or 1 gene per genome in the cluster. In our case, we included paralogues in the same cluster. This does not alter the shape of the distribution very much. There is still a U shape with a large peak at *k* = 1 and a smaller peak of core genes in all genomes. The infinitely-many genes model, introduced by Baumdicker et al. [8], predicts the shape of the spectrum under the assumption that each type of gene is introduced into the set of genomes only once, that it can spread to new genomes as the lineages of genomes divide, and that it can then be deleted independently from any genomes. This model qualitatively explains the U-shaped distribution, and we are currently developing models of this type in order to more quantitatively fit the *G*(*k*) distribution in our data. Baumdicker et al. [8] assume that the genealogical tree relating the genomes in the sample can be described by a coalescent tree, which is what we would expect for neutral evolution within a species. It is not yet clear whether this can be applied to describe the gene frequency spectrum across species (e.g. all Bacteria or all Archaea), as the tree arising from speciation may not resemble a coalescent tree. A quantity related to *G*(*k*) is the distribution of the total number of genes in a cluster. Enright et al. [10] plotted this for genes in MCL-derived clusters, and found that it was approximately a power law over quite a wide range of *n.* Developing a good quantitative model for this would require a combination of a branching process that generates the tree of genomes and a duplication/deletion model to describe gene families in each lineage.

The mean family size can be defined in two ways: either within a cluster, , or within a genome, ***.*** Comparison of  for different clusters revealed that more widespread clusters (i.e. larger *k*) tend to have larger families. This makes sense for genes that are beneficial. A gene that was beneficial in the genome in which it originated would remain in that genome for long enough to be duplicated and for long enough for a speciation to occur. Hence beneficial genes are more likely to have both paralogues and orthologues. The converse of this argument is that genes that are not beneficial in the genome in which they arise are likely to be deleted rapidly before duplication or speciation. It thus makes sense that  is very close to 1 for ORFan clusters with *k* = 1. The fact that *G*(1) is so large shows that there is a high rate of innovation of new gene families in individual lineages, and suggests that most of these families are not useful enough to be retained over long times.

In future work we will examine the dynamical aspects of gene family evolution to further investigate the process of gene innovation and to distinguish between innovation via evolution within a lineage and innovation via horizontal transfer from a genome outside the current data set. Horizontal gene transfer (HGT) clearly plays a role in bacterial genome evolution and one of our future goals is to understand this in a quantitative way. However, the stationary distribution of genome families in individual genomes is not instructive regarding HGT because it is not possible to distinguish what fraction of the innovation rate represents origin within the lineage and what fraction represents insertion of a gene by HGT. In our model we assume that the rate of gain of additional genes in a family is proportional to the number of genes already present. Interpreted as gene duplication, this means that genes in larger families are more frequently duplicated. However, this could also be interpreted as HGT if the majority of horizontal transfer events are between closely related organisms that form an effective bacterial “species” via frequent exchange of genes. In this case the number of genes in the family will be similar in all the genomes from which transfer is occurring, and the rate of gain of a gene by HGT will be proportional to the number of genes already present. Along these lines, Treangen and Rocha [32] have recently reported that the majority of the gains of additional members of multi-gene families occur by HGT rather than gene duplication. However, it is difficult to reconcile their findings with the observation that the majority of gene families are only found in a small number of genomes, which would not be the case if there were frequent repeated transfers of members of the same gene family. If there is a high diversity of genes in the pool from which genes can be gained by HGT, as suggested by the large numbers of singletons observed even in closely related organisms, then it is highly unlikely that a gene in the same gene cluster will be gained more than once. Together with our forthcoming phylogeny-based model, a more careful accounting of gene gains *and losses* will be required to reconcile these discrepancies.

Comparison of  for different genomes revealed that larger genomes have larger mean family sizes as well as larger number of families in total. This raises the question of why some genomes are larger than others among Bacteria and Archaea. The genomes of these organisms appear relatively streamlined, in the sense that there are relatively few non-coding regions and repetitive sequences in comparison to Eukaryotic genomes. This suggests that there is a high rate of deletion of junk DNA or that there is a cost to the replication of excess DNA or that there is a cost to possession of genes that do not produce beneficial proteins. It is possible that larger genomes are larger because these organisms occupy niches where a more diverse set of genes is necessary or beneficial to the organism. On the other hand, it could be that larger genomes get larger simply because the rate of deletion of non-functional genes is lower in these species or because the cost of non-functional genes is less, so that the genome can tolerate a higher proportion of genes that are not beneficial. This latter explanation fits with our observation that there is a higher proportion of duplicate genes in larger genomes (Fig. 3b). In contrast, the smallest genomes contain small numbers of large gene families. This suggests that there is a rapid rate of deletion of non-beneficial genes and significant selection against retention of these genes. Looking at gene gain and loss in a dynamical phylogenetic model would again help to clarify this, because one could estimate rates of gene gain and loss in different lineages.

## Authors' contributions

REC wrote the programs for data analysis, analyzed the data and wrote the paper. HM wrote the scripts for running BLAST and creating the gene clusters. PGH designed the study, developed the mathematical models and wrote the paper.

## Competing interests

The authors declare that they have no competing interests.
